# Effect of Dietary Nutrient Density on Small Intestinal Phosphate Transport and Bone Mineralization of Broilers during the Growing Period

**DOI:** 10.1371/journal.pone.0153859

**Published:** 2016-04-21

**Authors:** Jianhui Li, Jianmin Yuan, Zhiqiang Miao, Zhigang Song, Yu Yang, Wenxia Tian, Yuming Guo

**Affiliations:** 1 College of Animal Science and Veterinary Medicine, Shanxi Agricultural University, Shanxi 030801, China; 2 State Key Laboratory of Animal Nutrition, College of Animal Science and Technology, China Agricultural University, Beijing 100193, China; 3 Department of Animal Science, Shandong Agricultural University, Taian, Shandong 271018, China; Georgia Regents University, UNITED STATES

## Abstract

A 2 × 4 factorial experiment was conducted to determine the effects of dietary nutrient density on growth performance, small intestinal epithelial phosphate transporter expression, and bone mineralization of broiler chicks fed with diets with different nutrient densities and nonphytate phosphorus (NPP) levels. The broilers were fed with the same starter diets from 0 to 21 days of age. In the grower phase (day 22 to 42), the broilers were randomly divided into eight groups according to body weight. Relatively high dietary nutrient density (HDND) and low dietary nutrient density (LDND) diets were assigned metabolic energy (ME) values of 3,150 and 2,950 kcal/kg, respectively. Crude protein and essential amino acid levels were maintained in the same proportion as ME to prepare the two diet types. NPP levels were 0.25%, 0.30%, 0.35%, and 0.40% of the diets. Results showed that a HDND diet significantly increased the body weight gain (BWG) of broilers and significantly decreased the feed conversion ratio and NPP consumed per BWG. HDND significantly decreased tibial P content of the broilers. Conversely, mRNA expression of NaPi-IIb and protein expression of calbindin were significantly increased in the intestine of broilers fed a HDND diet. HDND also increased vitamin D receptor (VDR) expression, especially at a relatively low dietary NPP level (0.25%). The mRNA expression of NaPi-IIa in the kidneys was significantly increased at a relatively low dietary NPP level (0.25%) to maintain P balance. Tibial P, calcium, and ash content were significantly decreased, as were calbindin and VDR expression levels in the intestine at a low NPP level. Therefore, HDND improved the growth rate of broilers and increased the expression of phosphate and calcium transporter in the small intestine, but adversely affected bone mineralization.

## Introduction

The genetic evolution of broilers accelerated from 1957 to 2001, resulting in the number of days to market of commercial broilers decreasing from 120 to 42 days, while the average weight had doubled and the average feed conversion ratio (FCR, feed to gain), an important economic indicator of animal breeding programs, had improved from 3 to 1.7 [1]. However, although selective pressure increases the growth rate of broiler chickens, leg weakness also inadvertently evolves. A reduction in growth rate can improve gait score (GS), achieve good overall mobility, and yield a low incidence of skeletal disease [2]. However, the reduced occurrence of varus–valgus deformities in slow-growing chicks is unlikely related to improvements in the structure and composition of bone tissue. Likewise, the reduced growth rate of broiler chickens with a low energy diet improbably improves cortical bone quality [3]. Hence, reports have demonstrated contradicting effects of growth rate on bone development.

Intestinal absorption, renal excretion, and re-absorption of phosphate are essential for whole body phosphorus homeostasis. Sodium phosphate-mediated transcellular transfer is the dominant pathway of intestinal inorganic phosphate absorption, in which type IIb sodium-coupled phosphate transporter plays a major role [4]. In calcium-phosphorus regulatory systems, the activities of 25-hydroxyvitamin D3-1a-hydroxylase, intestinal calbindin, plasma phosphorus, and 1,25(OH)_2_D_3_ are significantly affected by different growth rates [5]. For instance, a high level of the type IIb sodium phosphate co-transporter is observed in high-growth rate broilers induced by high dietary nutrient density in the starter period [6]. Dietary calcium (Ca) and phosphorous (P) metabolism and requirements of fast-growing chickens are affected by age [7], as are densitometric and biochemical indices of broiler tibias [8, 9]. Nevertheless, studies to the best of our knowledge have rarely described the effects of growth rate on intestinal phosphate absorption of broilers during the growing period.

In order to reveal changes in intestinal phosphate absorption and P consumption affected by the growth rate of broilers during the growing period, the effects of changes in growth rate induced by dietary nutrient density on the expression of intestinal type IIb sodium phosphate co-transporter and bone mineralization were investigated.

## Materials and Methods

### Animals and Diets

The study protocol was approved and performed in accordance with the Guidelines for Experimental Animal Welfare of China Agricultural University (Beijing, China). A total of 480 one-day-old male Arbor Acres Plus broilers (Beijing Arbor Acres Poultry Breeding Company, Beijing, China) were selected and fed with the same diet until the age of 3 weeks. At this age, the broilers were weighed individually and grouped in 40 unit cages in terms of similar body weights. Five replicate pens containing six broilers each were prepared. The broilers were raised in battery cages that were each equipped with a feeder and water supply. The broilers were allowed access to mashed feed and water *ad libitum*. The temperature was initially maintained at 34°C when the broilers were aged 1 to 3 days and then gradually decreased to room temperature. The photoperiod was set at 24 h of light. At 42 days of age, the body weight, feed intake, and FCR of the broilers in each pen were measured. No mortality was recorded during the experimental period.

Eight diets were prepared in a 2 × 4 factorial experiment with two levels of nutrient densities at four non-phytate phosphorus (NPP) levels (Table 1). Two different nutrient density diets, namely, a high dietary nutrient density (HDND) and low dietary nutrient density (LDND) diets, were assigned metabolic energy (ME) values of 3150 and 2950 kcal/kg, respectively. Crude protein and essential amino acid levels were maintained in the same proportion as the ME values recommended by the Chinese Chicken Feeding Standard Requirements (NY/T 33–2004). The NPP levels for the HDND and LDND diets were 0.25%, 0.30%, 0.35%, and 0.40% of the diet. Each of the eight diets was fed to the six 3-week-old male Arbor Acres Plus broilers in the five replicate pens. Phytate and nonphytate phosphorus contents were determined.

**Table 1:** Composition of broiler grower diets formulated with different dietary nutrient density and phosphorus levels.

	Dietary Nutrient Density	
Dietary Ingredients (%)	High	Low
Corn	55.98	63.33
Soybean meal	30.08	29.13
Corn gluten meal	4.50	2.00
Soybean oil	5.55	1.69
Salt	0.30	0.30
Trace mineral premix^1^	0.20	0.20
Vitamin premix^2^	0.03	0.03
DL-methionine	0.09	0.09
L-lysine HCl	0.06	0.01
Choline chloride	0.30	0.30
Aureomycin	0.06	0.06
Antioxidant	0.03	0.03
Various^3^	2.82	2.83
Total	100.00	100.00
Nutrient composition		
Metabolizable energy (kcal/kg)	3150	2950
Crude protein (%)	20.32	19.03
Calcium (%)	0.56	0.56
Nonphytate phosphorus^4^ (%)	0.25	0.25
Lysine (%)	1.02	0.95
Methionine (%)	0.41	0.38
Tryptophan (%)	0.22	0.21
Threonine (%)	0.76	0.72

^1^Nutrients per kilogram of diet: Cu (from CuSO4 ·5H2 O), 16 mg; Fe (from FeSO4 ·7H2 O), 80 mg; Zn (from ZnSO4 ·7H2 O), 110 mg; Mn (from MnSO4·H2 O), 120 mg; I (from Ca(IO3) 2 ·H2 O), 1.5 mg; Co (from CoCl2·6H2 O), 0.5 mg; Se (from organic selenium), 0.3 mg.

^2^Nutrients per kilogram of diet: vitamin A, 12,500 IU; vitamin D3, 3,000 IU; vitamin E, 25 mg; vitamin K3, 2.5 mg; thiamin, 2.5 mg; riboflavin, 8 mg; vitamin B12, 0.025 mg; folic acid, 1.25 mg; niacin, 37.5 mg; pantothenic acid, 12.5 mg; biotin, 0.125 mg.

^3^Variable amounts of dicalcium phosphate, limestone, or Maifanite.

^4^Dietary non-phytate phosphorus (NPP) level to each dietary nutrient density was 0.25%, 0.30%, 0.35%, or 0.40%.

### Measurements

#### Growth performance

At 42 days of age, the body weight of each broiler and the weight of the remaining feed in the trough of each replicate cage were determined after 4 h of feed withdrawal. Live body weight, feed intake, and FCR from day 21 to 42 were calculated. The health status of all broilers in each pen was checked daily.

#### Tissue sampling and preparation

At the age of 42 days, one broiler per cage was randomly selected and sacrificed through cervical dislocation. The duodenum segment (spanning the distal end of the gizzard to 1 cm of the distal end of the bile duct) was isolated immediately from the gastrointestinal tract. The digesta was thoroughly washed out of the duodenum with 1‰ cold diethylpyrocarbonate-treated water. Duodenal mucosa was scraped off on ice with a glass microscope slide and quickly frozen in liquid nitrogen for subsequent determination of protein and RNA expression. The kidneys were also removed for the determination of RNA expression. The left tibias of the individual broilers were excised, sealed in plastic bags, and stored at −30°C for further analysis [10].

#### Tibial strength, tibia ash content, and Ca and P concentration

The left tibia was de-fleshed manually and the patella was removed. These body parts were then air-dried for 24 h at room temperature and defatted to determine bone ash content, and concentrations of Ca and P. Dry-defatted tibias were ashed in a muffle furnace at 550°C for 16 h and tibia ash was measured on the basis of the percentage of dry weight. Ca and P contents were determined through EDTA titration and ammonium metavanadate colorimetry, respectively, and values were presented on the basis of dry-defatted weight [10].

#### Total RNA extraction, reverse transcription, and real-time PCR

Total RNA was extracted from the duodenal mucosa and kidney of 42-day-old broilers using the SV Total RNA Isolation System (Z3100; Promega Corporation, Madison, WI, USA) in accordance with the manufacturer’s instructions. The resulting extracts were re-suspended in diethylpyrocarbonate-treated water. The concentration and quality of the extracted RNA were determined through absorbance determination at 260 nm and agarose gel electrophoresis, respectively [6]. Afterward, 1.0 μg of total RNA was reverse-transcribed into single-stranded cDNA with AMV reverse transcriptase and an Oligo (dT)_15_ primer in the presence of recombinant RNasin ribonuclease inhibitor (A3500; Promega Corporation). The mRNAs of NaPi-IIb and NaPi-IIa were subjected to real-time PCR with β-actin as an internal control standard. The primers used in this experiment and the lengths of the obtained PCR products are shown in Table 2. Real-time PCR was conducted using an ABI 7500 fluorescent quantitative PCR system with RealSuper Mixture (with ROX) (CW0767, Beijing ComWin Biotech Co., Ltd., Beijing, China). The following real-time PCR protocol was applied: 95°C for 4 min; 40 cycles of 95°C for 15 s, 60°C for 60 s; and 60°C to 95°C. Melting curve analysis was then performed. Each gene was amplified in triplicate. Standard curves were also run to determine amplification efficiency. Relative standard curve methods were used to quantify gene expression. The results are expressed as the ratio of target gene mRNA to β-actin mRNA [11].

**Table 2:** Oligonucleotide PCR primers.

Gene	GenBank accession	Orientation	Primer sequence (5ʹ to 3ʹ)	Predicted size (bp)
NaPi-IIb	NM_204474.1	Forward	CTTTTACTTGGCTGGCTGGAT	148
		Reverse	AGGGTGAGG**G**GATAAGAACG	
NaPi-IIa	AF297188.1	Forward	CCGCACCTCCCCAGACT	100
		Reverse	GTTGTGGAGGATCCCAATGC	
β-Actin	NM_205518.1	Forward	AACACCCACACCCCTGTGAT	100
		Reverse	TGAGTCAAGCGCCAAAAGAA	

#### Western blot analysis for vitamin D receptor and calbindin protein

The duodenal brush-border membrane vesicles were homogenized and centrifuged at 10,000 ×*g* for 5 min at 4°C and the resulting supernatants were stored at -80°C for further use. Protein concentration was determined using the Bradford assay [12]. The samples of brush-border membrane vesicles were placed in Laemmli buffer (Sigma–Aldrich, St. Louis, MO, USA) for 5 min to induce protein denaturation. Afterward, 50 mg of brush-border membrane vesicle proteins were loaded onto each lane, separated on a 4% polyacrylamide gel, and subsequently transferred onto a polyvinylidene difluoride membrane for 2 h. The polyvinylidene difluoride membrane was then probed for the presence of vitamin D receptor and calbindin by incubating with primary antibody diluted to 1:1000 for at least 1 h. Then, the membrane was washed in TBST buffer (Tris-buffered saline, 0.1% Tween 20) and incubated with secondary antibody conjugated with horseradish peroxidase (1:5000, Bio-Rad laboratories, Hercules, CA, USA). Immunoblots were visualized on an X-ray film through chemiluminescence (Pierce Protein Research Products, Rockford, IL, USA). Optical density-calibrated images were analyzed using AlphaEase Stand Alone software (Alpha Innotech Corporation, Santa Clara, CA, USA) as described elsewhere [13].

### Statistical Analysis

Replicate means were used as the experimental unit in statistical analysis. The results were analyzed using the GLM procedure included with the SPSS ver. 16.0 software package (IBM-SPSS, Inc., Chicago, IL, USA) to estimate the main effects. When the main effect of a treatment was significant, the differences between the means were assessed using Duncan’s multiple range analysis. The mean was considered significantly different at *p* < 0.05.

## Results and Discussion

### Broiler performance

The growth, livability, and FCR of broilers has accelerated from 1957 to 2001 [1]. Dietary nutrient density is a dominant factor affecting the growth rate of broilers. In this study, body weight gain (BWG) was significantly increased (*p* < 0.05), but the FCR and NPP consumed per BWG were significantly decreased (*p* < 0.01), as was feed intake (0.1 > *p* > 0.05) among broilers fed a HDND diet (Table 3). Enteral nutrition can enhance oxidative phosphorylation and ATP synthesis [14]. Moreover, duodenal mitochondria play an important role in the phenotypic expression of feed efficiency in broilers [15]. The mitochondria produce 90% of cellular energy [16]; thus, some of the variations in broiler growth performance and feed efficiency in this study may have been due to differences in mitochondrial functions, thus these differences should be further investigated. The NPP consumed per BWG of the broilers during the growing period was significantly decreased by a HDND diet, the same as in the starter period [6]. Although a HDND diet can retain a specific amount of phosphorus necessary to promote weight gain, a low amount of phosphorus was retained in the bone (Table 4); thus, excess phosphorus may be used to stimulate abdominal fat deposition [15,16].

**Table 3:** Growth performance of broilers as influenced by different dietary nutrient density and phosphorus levels.

Dietary nutrient density	Non-phytate phosphorus (%)	Weight gain (g)	Feed intake (g)	Feed conversion	NPP consumed per gain (g/kg)
Low	0.25	1.09	2.33	2.142^d^	5.35^b^
	0.30	1.11	2.33	2.106^d^	6.32^d^
	0.35	1.13	2.32	2.036^c^	7.21^f^
	0.40	1.20	2.39	1.994^b^^c^	7.97^h^
High	0.25	1.18	2.29	1.936^a^^b^	4.84^a^
	0.30	1.21	2.31	1.922^a^^b^	5.76^c^
	0.35	1.18	2.28	1.948^a^^b^	6.81^e^
	0.40	1.23	2.33	1.894^a^	7.58^g^
SEM		0.01	0.011	0.007	0.032
Main effects					
Dietary nutrient density					
Low		1.13^a^	2.34	2.070^b^	6.71^b^
High		1.20^b^	2.30	1.925^a^	6.25^a^
Non-phytate phosphorus					
0.25		1.14	2.31	2.015	5.07^a^
0.30		1.17	2.34	2.001	6.00^b^
0.35		1.18	2.34	1.983	6.97^c^
0.40		1.20	2.37	1.971	7.93^d^
*p*-value					
Dietary nutrient density		0.018	0.070	<0.001	<0.001
Non-phytate phosphorus		0.229	0.221	0.174	<0.001
Dietary nutrient density × Non-phytate phosphorus		0.479	0.664	0.006	0.041

^a–h^Within a column, values not sharing a common superscript letter are significantly different (*p* < 0.05).

**Table 4:** Mineral composition of broiler tibias as influenced by different dietary nutrient density and phosphorus levels.

Dietary nutrient density	Non-phytate phosphorus (%)	Tibia P (%)	Tibia Ca (%)	Tibia ash (%)
Low	0.25	8.44	16.94	48.36
	0.30	8.52	17.52	50.19
	0.35	9.25	18.61	53.16
	0.40	9.11	18.54	53.37
High	0.25	7.20	16.43	47.77
	0.30	8.35	17.72	51.00
	0.35	8.92	17.95	51.66
	0.40	9.04	18.25	52.89
SEM		0.092	0.097	0.240
Main effects				
Dietary nutrient density				
Low		8.83^b^	17.90	51.27
High		8.38^a^	17.59	50.83
Non-phytate phosphorus				
0.25		7.82^a^	16.68^a^	48.07^a^
0.30		8.44^b^	17.62^b^	50.59^b^
0.35		9.08^b^	18.28^b^^c^	52.41^c^
0.40		9.08^b^	18.40^c^	53.13^c^
*p*-value				
Dietary nutrient density		0.021	0.118	0.367
Non-phytate phosphorus		<0.001	<0.001	<0.001
Dietary nutrient density × Non-phytate phosphorus		0.127	0.452	0.427

^a–c^Within a column, values not sharing a common superscript letter are significantly different (*p* < 0.05).

The NPP level did not significantly affect BWG, feed intake, or FCR of the broilers (*p* > 0.05). However, the NPP consumed per BWG was significantly increased by high dietary NPP levels, as in the starter period [6]. This observation also indicated that a high NPP diet promoted highly efficient phosphorus utilization by broilers [17]. The nutrient density and NPP level were significantly associated with feed conversion (*p* < 0.01) and NPP consumed per BWG of the broilers (*p* < 0.05). The FCR was greatly decreased by a HDND diet at a high NPP level. Thus, the ratio of nutrient density to phosphorus should be considered in feed formulation.

### Tibia mineral composition

Food restriction can affect bone development; however, contradictory results have been presented. For instance, Leterrier and Rose [18] reported no improvement in bone quality by reducing the growth rate of broiler chickens with a low-energy diet. Furthermore, the reduced occurrence of varus–valgus deformities in slow-growing chicks is unlikely related to the improvement in bone structure and composition. Conversely, Brickett et al. [19] demonstrated that a reduced growth rate can yield a higher GS, more efficient overall bird mobility, and lower incidence of skeletal disease than an increased growth rate. Bruno et al. [20] also indicated that food restriction with 40% of *ad libitum* intake can reduce bone length and width without affecting bone weight. Our study partially verified the assumption that the bone mineral content of broilers remained unchanged as growth rate varied, and dietary nutrient density did not significantly affect tibia ash or Ca content (*p* > 0.05). By contrast, the tibia P content of broilers was significantly increased by a LDND diet, but was decreased by a HDND diet. Therefore, the incidence of skeletal disease of low-growth rate chickens is low [19].

In broiler chicks, tibia ash, BMC, and bone mineral density are more sensitive indicators of dietary Ca and P concentrations than shear force [21]. However, bone shear force was not affected by the treatments (data not shown), while the NPP level significantly affected tibial P and Ca concentrations, and tibia ash content (*p* < 0.01). In particular, tibia P, tibia Ca, and tibia ash increased linearly as the NPP level in the diet increased. This finding is consistent with our previous results [22]. The interaction between nutrient density and NPP level was not significantly associated with tibia mineral composition (*p* > 0.05).

### Phosphorus and calcium transporter expression in the duodenum and kidneys

The expression of phosphorus and calcium transporter and the expression of vitamin D receptor in the duodenum of broilers were significantly affected by dietary nutrient density (Table 5). Compared with LDND, a HDND diet significantly improved mRNA expression of intestinal NaPi-IIb in the duodenum (*p* < 0.05). Likewise, protein expression of calbindin and VDR were significantly (*p* < 0.01) enhanced in the duodenum of broilers fed a HDND diet. By contrast, mRNA expression of NaPi-IIa in the kidney was not affected by dietary nutrient density. The results in the growing and rearing periods are consistent with those in the starter period, and the similarity of these findings may be attributed to the effects of metabolic acidosis generated by high-protein diets. Intestinal sodium phosphorus-IIb expression can also be stimulated during metabolic acidosis to provide phosphate and prevent excessive liberation of phosphate from the bone [23]. Moreover, long-term calorie restriction affects intestinal transport of phosphate and remarkably reduces expression of NaPi-IIb, which responds to changes in energy levels in enterocytes [24]. The gene expression and uptake of the intestinal sodium-phosphate co-transporter may be related to either fructose or glucose metabolism in the intestine [25, 26], and these processes may also be regulated by epidermal growth factor (EGF) [27]. Our study also verified that EGF secretion was reduced and NaPi-IIb expression was increased in broilers fed a HDND diet [28]. EGF is a small protein that can act on the NaPi-IIb promoter in a reverse direction, in which down regulation of promoter function is mediated by EGF-activated PKC/PKA and MAPK pathways [27].

**Table 5:** Phosphate and calcium transport in broilers in response to different dietary nutrient density and phosphorus levels.

Dietary nutrient density	Non-phytate phosphorus (%)	Duodenum			Kidney
		NaPi-IIb mRNA	Calbindin protein	VDR protein	NaPi-IIa mRNA
Low	0.25	0.835	0.400	0.108^a^	1.48^a^^b^^c^
	0.30	0.826	0.525	0.387^b^	2.21^b^^c^
	0.35	0.623	0.601	0.482^b^	1.18^a^^b^
	0.40	1.282	0.727	0.633^c^	2.50^c^
High	0.25	1.277	0.824	0.762^c^^d^	3.96^d^
	0.30	1.361	1.189	1.008^e^	1.11^a^^b^
	0.35	0.846	1.019	0.797^d^	1.36^a^^b^
	0.40	1.244	0.933	0.691^c^^d^	0.52^a^
SEM		0.067	0.029	0.016	0.225
Main effects					
Dietary nutrient density					
Low		0.891^a^	0.563^a^	0.402	1.843
High		1.182^b^	0.991^b^	0.814	1.738
Non-phytate phosphorus					
0.25		1.056	0.612^a^	0.435	2.72
0.30		1.094	0.857^b^	0.698	1.66
0.35		0.734	0.810^b^	0.639	1.27
0.40		1.263	0.830^b^	0.662	1.51
*p*-value					
Dietary nutrient density		0.045	<0.001	<0.001	0.677
Non-phytate phosphorus		0.074	0.025	<0.001	0.001
Dietary nutrient density × Non-phytate phosphorus		0.480	0.075	<0.001	<0.001

^a–e^Within a column, values not sharing a common superscript letter are significantly different (*p* < 0.05).

Approximately 99% of body Ca is stored in the skeleton. In our study, the low tibia ash and bone mineral content induced by 0.25% dietary non-phytate phosphorus may have been associated with low protein expression of calbindin and VDR in the intestine, which is highly related to bone health through the regulation of parathyroid hormone (PTH) [29]. The mRNA of intestinal sodium phosphorus-IIb is transcriptionally regulated by vitamin D receptor [30].

Nutrient density and NPP levels in the diet were significantly associated with the protein expression of VDR in the duodenum and with the RNA expression of NaPi-IIa in the kidneys (*p* < 0.01). Protein expression of VDR was remarkably increased in broiler fed a HDND diet with a relatively low dietary NPP level (0.25%). Vitamin D_3_ synthesis is stimulated by PTH, which increases the activity of 25-hydroxyvitamin D3-1a-hydroxylase by acting on the promoter of the 1a-hydroxylase gene [31]. 1α-Hydroxy cholecalciferol can increase not only tibia ash and tibia strength, but also mRNA expression of intestinal sodium phosphorus-IIb [11]. mRNA expression of NaPi-IIa in the kidneys was significantly decreased by HDND at a relatively high dietary NPP level (0.4%). By contrast, mRNA expression of NaPi-IIa in the kidneys was significantly increased at a relatively low dietary NPP level (0.25%) to maintain phosphorus balance.

## Conclusions

In conclusion, HDND improved the growth rate of broilers and increased expression of the P and Ca transporter in the small intestine, although a HDND diet adversely affected bone mineralization.
